# A rare case of endo-bronchial lipoma presenting with vague cardiac symptoms

**DOI:** 10.1093/jscr/rjae703

**Published:** 2025-01-15

**Authors:** Zoha Asghar, Muhammad Mehdi Irfani, Alizeh Fatimi, Abdul Ahad Sohail, Saulat Hasnain Fatimi

**Affiliations:** Department of Surgery, Aga Khan University Hospital, 74800, Karachi, Pakistan; Section of Cardiothoracic Surgery, Aga Khan University Hospital, 74800, Karachi, Pakistan; Aga Khan University, 74800, Karachi, Pakistan; Section of Cardiothoracic Surgery, Aga Khan University Hospital, 74800, Karachi, Pakistan; Section of Cardiothoracic Surgery, Aga Khan University Hospital, 74800, Karachi, Pakistan

## Abstract

Endobronchial lipoma is an extremely rare benign tumor, accounting for 0.1%–0.5% of all lung tumors. This case report presents a patient diagnosed with endobronchial lipoma, a condition that can lead to significant bronchial obstruction and subsequent parenchymal damage. Patients usually exhibit symptoms including cough, dyspnea, and recurrent respiratory infections, which initially mimic more common pulmonary conditions such as chronic obstructive pulmonary disease. However, in our case patient presented with non-specific cardiac symptoms. Diagnosis was confirmed through bronchoscopy, revealing a well-circumscribed mass obstructing the bronchus. Due to severe parenchymal inflammation, treatment involved left-sided thoracotomy and upper lobectomy which successfully relieved the obstruction and alleviated the patient’s symptoms. Histopathological examination identified the tumor as a lipoma. This case underscores the importance of considering endobronchial lipoma in differential diagnoses for patients with unexplained cardiac or respiratory symptoms, as early detection and treatment can prevent significant complications.

## Introduction

Endobronchial lipomas are rare benign tumors, accounting for only 0.1%–0.5% of all bronchial tumors. Originating from the adipose tissue within the bronchial wall, these tumors can lead to significant bronchial obstruction and subsequent parenchymal damage [1, 2]. Clinical manifestations of endobronchial lipomas are varied and may include symptoms such as shortness of breath, recurrent pneumonia, atelectasis, and hemoptysis, all resulting from the obstructive effects of the tumor [3]. Due to these nonspecific symptoms, endobronchial lipomas are often misdiagnosed as more common obstructive pulmonary conditions like chronic obstructive pulmonary disease (COPD) or asthma [4]. Histologically, endobronchial lipomas are characterized by numerous uniform adipocytes. They tend to occur more frequently on the right side of the bronchial tree and are more prevalent in males [5].

We report a rare case of an endobronchial lipoma in a middle-aged female, who presented with non-specific cardiac symptoms due to a complete obstruction of the left upper lobe.

## Case presentation

**Figure 1:**
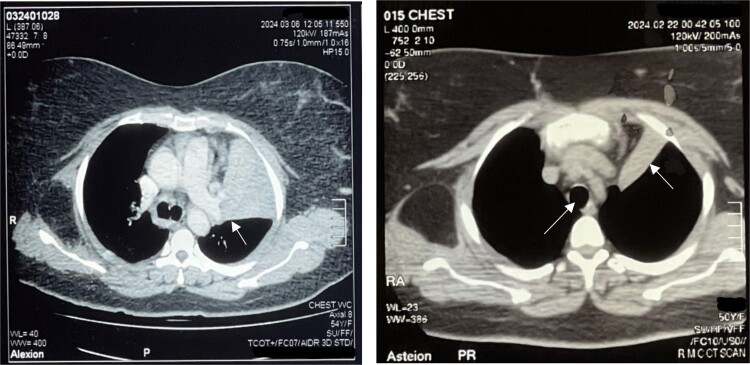
Left: Right main bronchus shown by black arrowhead and inflamed lung parenchyma shown by arrowhead. Right: Trachea and collapsed lung on CT scan (white arrowhead).

A 50-year-old female, with hypertension and asthma, presented with left shoulder pain and chest heaviness persisting for a month. She denied substance use and had no family history of cardiovascular disease. Initially suspected of coronary syndrome, a negative cardiac workup was conducted at an outside hospital. Chest auscultation revealed normal findings, and a prior CT scan indicated a large fat-density lesion measuring approximately 9.1 × 6.9 × 9.3 mm under the scapula, with consolidative opacities in the left lung lingular segments suggestive of collapse consolidation (Fig. 1).

**Figure 2:**
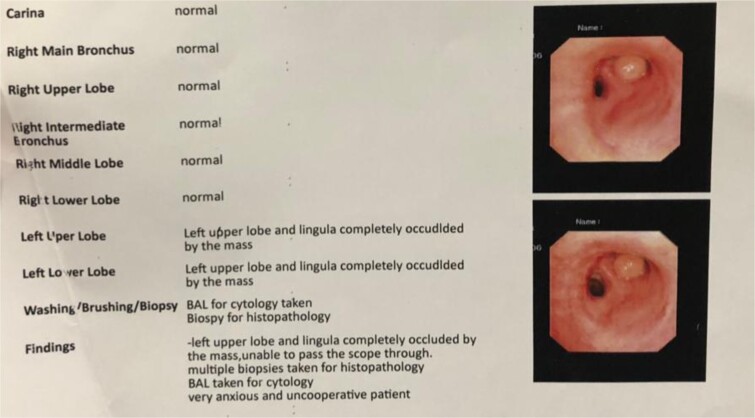
Bronchoscopy report confirming a mass completely occluding the left upper lobe and lingula (Fig. 3). Endobronchial lipoma along the left main bronchus (white arrowhead).

Bronchoscopy confirmed complete occlusion of the left upper lobe and lingula by a mass (Fig. 2), and subsequent CT with contrast revealed luminal obliteration of the left upper lobe bronchus by a long linear fat-density structure. A right posterolateral chest wall lipoma was also noted. Bronchoalveolar lavage at our center showed bronchial mucosa with moderate lymphoplasmacytic infiltrates, with fungal culture positive for Aspergillus terreus and bacterial culture positive for *Staphylococcus aureus*. GeneXpert testing excluded tuberculosis.

**Figure 3:**
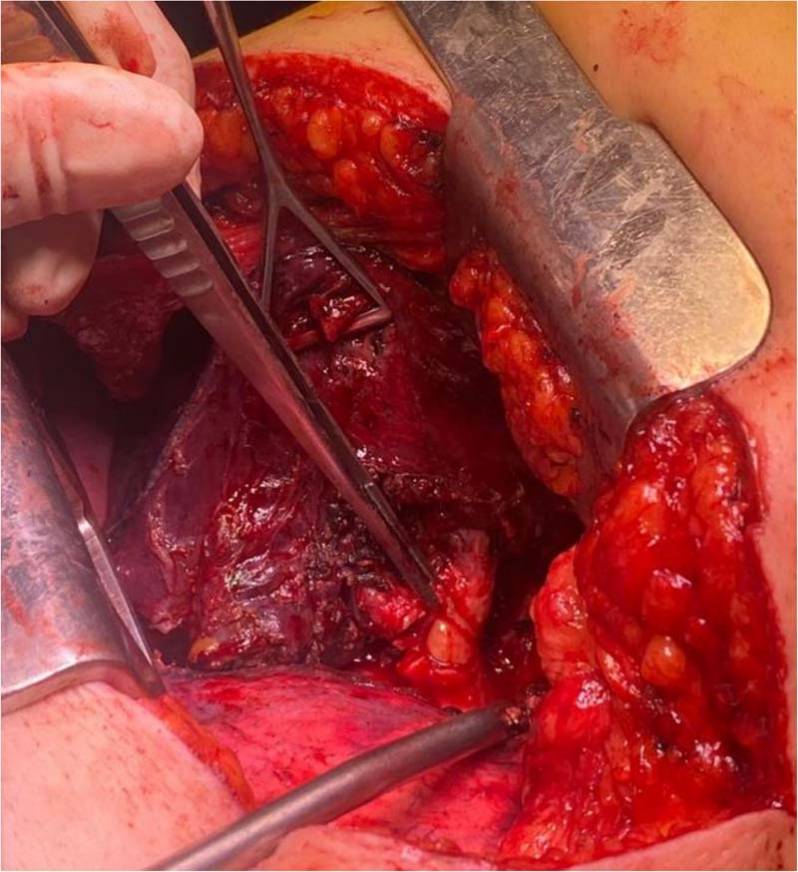
Endobronchial lipoma along the left main bronchus (arrowhead).

**Figure 4:**
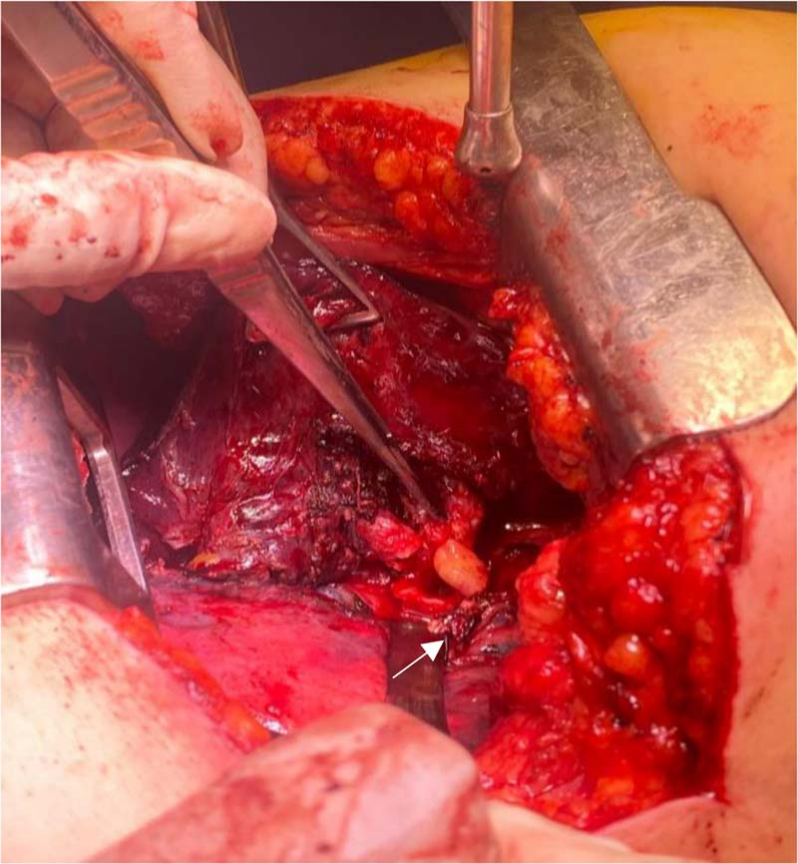
Endobronchial lipoma along the left main bronchus (arrowhead).

The patient underwent left-sided thoracotomy and upper lobectomy due to severe parenchymal inflammation and necrosis of the left upper lobe, with histopathology confirming a well-circumscribed adipocytic lesion consistent with lipoma measuring ~9.1 × 6.9 × 9.3 mm in the bronchus (Figs 3 and 4). Adjacent lung tissue exhibited organizing pneumonia and bronchiectasis. Post-operatively, she recovered well and was discharged home in stable condition on the fourth day.

## Discussion

Endobronchial lipomas, rare benign tumors constituting only 0.1% of all lung tumors [2], predominantly affect males aged 50–70 years, demonstrating a significant male predominance with a ratio of 45:7 males to females [6]. Smoking and obesity are recognized as significant risk factors for their development [7, 8]. These tumors typically arise from peri-bronchial and submucosal fat tissue, with two-thirds occurring on the right side of the tracheobronchial tree. The presentation of a left upper lobe bronchus lipoma in a female patient, as observed in this case, represents a notable exception to this typical distribution [9, 10].

The patient initially presented with non-specific symptoms of left shoulder pain and chest heaviness, which prompted comprehensive evaluation including a contrast-enhanced CT scan of the lungs. This imaging modality revealed a characteristic fat tissue density within the left upper lobe bronchus, confirming the presence of the lipoma.

CT scans of the lungs are diagnostic, revealing characteristic fat tissue density without contrast enhancement [7]. This feature distinguishes endobronchial lipomas definitively from other pulmonary lesions such as fat-rich pulmonary hamartomas, which may include additional mesenchymal components [11]. While chest X-rays may show post-obstructive changes such as atelectasis or pneumonia in 80% of cases, their sensitivity for diagnosing endobronchial lipomas is limited (66%) [12].

Furthermore, the patient’s initial presentation with left shoulder pain and chest heaviness, devoid of typical respiratory symptoms, underscores the non-specific nature of clinical manifestations linked with endobronchial lipomas [13]. Such presentations often prompt investigations for cardiovascular etiologies, as evidenced by the negative cardiac workup in this case. This diagnostic pathway highlights the importance of considering less common pulmonary pathologies when differentiating non-specific thoracic symptoms.

Endobronchial lipomas are often diagnosed after symptoms emerge, thus potential complications such as post obstructive pneumonia and chronic lung inflammation may be present. To prevent distal lung damage caused by chronic obstruction, the primary treatment approach involves surgical or endoscopic resection [14]. Bronchoscopic treatment is now recommended as the initial therapy due to its ability to alleviate symptoms with minimal procedural risk compared to surgery [3]. However, the choice between surgical and bronchoscopic methods depends on several factors. Bronchoscopic resection is suitable for tumors confined within the bronchial tree and centrally located [15]. However, larger tumors, those extending beyond the bronchial wall, or those with a dumbbell shape on CT scans may not be suitable for endoscopic procedures. Additionally, if the benign nature of the tumor is uncertain or if it has caused severe and irreversible damage to the peripheral lung tissue, complete recovery may not be achievable through bronchoscopic means alone. In such cases, surgical options like lobectomy, wedge resection, or pneumonectomy may be preferred [16].

In our presented case, surgical resection in the form of left upper lobectomy was ultimately required due to severe parenchymal inflammation and necrosis. This decision highlights the critical role of surgical intervention in cases where endobronchial lipomas cause significant structural lung damage or fail to respond adequately to bronchoscopic management.

Prognostically, the recurrence rates of endobronchial lipomas post-resection are low, and the risk of malignant transformation is minimal. Complete surgical resection typically results in good outcomes, emphasizing the curative potential of timely and definitive management [12].

## Conclusion

Endobronchial lipomas, though rare and typically benign, can present with varying degrees of airway obstruction leading to diverse clinical symptoms. The management approach depends on individual patient characteristics, tumor anatomy, and the overall condition of the affected lung. Despite their rarity, the prognosis following resection is generally favorable, with low recurrence rates reported in previous case studies. However, due to the potential for relapse, some experts advocate for vigilant post-operative monitoring. In conclusion, the treatment of endobronchial lipomas should be tailored to each patient, emphasizing personalized care to achieve optimal outcomes and minimize the risk of recurrence.
